# The vast chasm in ChatGPT assisting in realistic surgery

**DOI:** 10.1097/JS9.0000000000000657

**Published:** 2023-08-09

**Authors:** Xinxin Sun, Jingbo Wei, Xiaojing Wang, Bing Wang, Zhixiang Fan, Shi Wan, Ke Zhang, Dongmei Huang, Qing Zhang

**Affiliations:** a Department of Obstetrics and Gynecology, The Second Affiliated Hospital of Zhengzhou University; b Department of Electrocardiogram, Henan Cancer Hospital, Zhengzhou, Henan, People’s Republic of China

*Dear Editor*,

With the generalization of ChatGPT in all walks of life, there is a debate on ChatGPT in assisting in surgery. Our point of view is more from the professional perspective in the real world.

Medicine mainly focuses on the diagnosis, treatment, and prevention of diseases, injuries, and disorders, which is the combination of science and practice in humans. It encompasses a wide range of knowledge, skills, and practices aimed at promoting health, alleviating suffering, and improving the well-being of individuals. The primary mission of medicine is to promote healing and provide compassionate care to individuals. Meanwhile, it is a holistic approach which recognizes the interconnectedness of physical, mental, and social aspects of health. Physicians strive to understand the whole person, considering their unique circumstances, cultural backgrounds, values, and preferences when making treatment decisions. Thus, ethical principles and patients’ rights are maintained inherent in medicine. Physicians prioritize patient autonomy, informed consent, confidentiality, and respect for cultural diversity. Physicians are expected to demonstrate professionalism, empathy, and cultural sensitivity in their interactions with patients and colleagues.

Medicine is grounded in evidence-based practice, which involves integrating the best available scientific evidence with clinical expertise and patient values. Physicians rely on research, clinical trials, and systematic reviews to guide their decision-making and ensure the most effective and safe treatments for their patients^1,2^.

Surgery is one type of medical treatment for patients. It is distinguished by its invasive nature, where incisions or access points are made in the patient’s body to gain direct access to the affected area. This allows surgeons to visualize and physically interact with the tissues or organs that require treatment. Patient safety is paramount in surgery. Surgeons and the surgical team follow strict protocols to minimize the risks associated with surgery, such as infection, bleeding, or complications. Safety measures include proper sterilization, monitoring vital signs, utilizing advanced technology, and adhering to established surgical guidelines. There are the following elements affecting the difficulty of one surgery.

Human anatomy can have significant variations among `individuals, making surgeries more challenging. Variations in anatomy can impact surgical approaches, increase the risk of complications, and require adaptations during the procedure. Surgeons must have a thorough understanding of anatomical structures, including their variations. The patient’s overall health and condition can influence the difficulty of a surgical procedure. Factors such as age, underlying medical conditions, obesity, previous surgeries, and anatomical variations can increase the complexity of the surgery and the associated risks. Surgeons must carefully assess and manage these factors to optimize patient outcomes. Surgeries can sometimes encounter unexpected findings or complications, which can increase the difficulty of the procedure. Surgeons must be prepared to adapt their approach, address complications promptly, and make real-time decisions to ensure patient safety and the best possible outcome. Emergency surgeries, such as those performed for trauma or acute conditions, often present unique challenges. Surgeons must make quick decisions, work under time constraints, and address complex injuries or life-threatening situations. Emergency surgeries require rapid assessments, prioritization of interventions, and the ability to handle unexpected complications. Thus, surgical skills are acquired through extensive training, practice, and experience. Surgeons need to develop excellent hand–eye coordination, dexterity, and spatial awareness to perform surgeries effectively. Advanced surgical techniques, such as minimally invasive procedures or robotic-assisted surgeries, may require additional training and expertise. Surgery can be emotionally and mentally demanding for surgeons. They must handle high-pressure situations, make critical decisions, and communicate effectively with patients, families, and the surgical team. Surgeons must also cope with the potential stress and emotional impact of performing complex and life-altering procedures. Surgeons must undergo extensive training and acquire specialized skills to perform surgical procedures effectively. Their expertise includes precise manipulation of tissues, organs, and other structures while ensuring minimal damage to surrounding healthy tissues and decreasing bleeding during the procedure.

Due to the invasive nature of the surgery, during and in the immediate postoperative period, anesthesia aims to eliminate or minimize pain. By blocking pain signals, anesthesia allows the surgical procedure to be performed without causing significant discomfort to the patient. Effective pain management during and after surgery is crucial for patient comfort, faster recovery, and reducing the risk of complications. Besides pain relief, anesthesia plays a critical role in inducing a reversible state of unconsciousness, muscle relaxation, and physiological stability in surgery by ensuring patient comfort, safety, and the successful completion of surgical procedures. Anesthesiologists evaluate the patient’s medical history, perform preoperative assessments, and determine the most appropriate anesthesia plan. During surgery, they closely monitor the patient’s vital signs, oxygen levels, heart rhythm, blood pressure, and depth of anesthesia to ensure the patient’s safety and adjust the anesthetic as needed. Anesthesia is tailored to each patient’s specific needs, considering factors such as age, overall health, medical history, allergies, and the nature of the surgical procedure. Anesthesiologists consider various factors when selecting the most appropriate anesthesia techniques, medications, and monitoring methods to optimize patient outcomes. After the surgical procedure, patients are closely monitored during the recovery phase in a specialized post-anesthesia care unit. Anesthesiologists or specialized nurses assess the patient’s recovery, manage any immediate postoperative pain or complications, and ensure the patient’s safe transition to a stable condition^3^.

Anesthesia is an integral part of surgical care, ensuring patient comfort, safety, and successful surgical outcomes. Anesthesiologists work collaboratively with the surgical team to provide individualized anesthesia care, optimize pain management, and maintain physiological stability throughout the perioperative period^4,5^.

Besides, nursing care in surgery is also crucial in providing comprehensive and patient-centered care throughout the perioperative period. Nurses play a vital role in supporting patients before, during, and after surgery, collaborating with the surgical team, and ensuring the well-being and safety of patients. Pre-operation, nurses assess patients’ medical history, perform physical examinations, and gather the necessary information to develop individualized care plans. They collaborate with other healthcare providers to ensure patients are appropriately prepared for surgery, including proper fasting, medication management, and completion of preoperative tests. Intra-operation, they ensure proper patient positioning, confirms patient identification and surgical site, and communicate essential information to the surgical team to prevent errors and ensure patient safety. Nurses assist in maintaining a sterile environment in the operating room. They follow strict infection control practices, including proper hand hygiene, donning sterile gowns and gloves, and preparing sterile equipment and supplies. They collaborate with the surgical team to prevent surgical site infections. Nurses assist surgeons by providing necessary instruments and supplies during the procedure. They anticipate the surgeon’s needs, handle specimens appropriately, and document surgical events accurately. Post-operation, nurses assess surgical incisions, monitor wound healing, and provide wound care as per surgical protocols. They ensure proper dressing application, assess for signs of infection, and educate patients on incision care to prevent complications. Nurses provide instructions to patients and families regarding postoperative care, medications, activity restrictions, and signs of complications. They ensure patients understand the importance of follow-up appointments and provide necessary resources for ongoing recovery and support. Nurses provide emotional support to patients and their families throughout the surgical process. They offer reassurance, address anxiety or concerns, and promote a calming and supportive environment^6^.

Nursing care in surgery is comprehensive and encompasses preoperative, intraoperative, and postoperative aspects. By providing skilled and compassionate care, nurses contribute significantly to positive patient outcomes, patient safety, and overall satisfaction during the surgical experience^7^.

Besides anesthesiologists and nurses, surgeons often collaborate with other healthcare professionals, such as radiologists and pathologists, to ensure comprehensive patient care. These collaborative efforts involve preoperative consultations, intraoperative support, and postoperative follow-up to optimize patient outcomes. It is a field of multidisciplinary treatment^8^ (Fig. 1). Surgery is a dynamic field that continuously evolves through medical research, technological advancements, and innovative techniques. New surgical approaches, tools, and technologies are developed to enhance surgical precision, minimize invasiveness, reduce recovery time, and improve patient outcomes.

**Figure 1:**
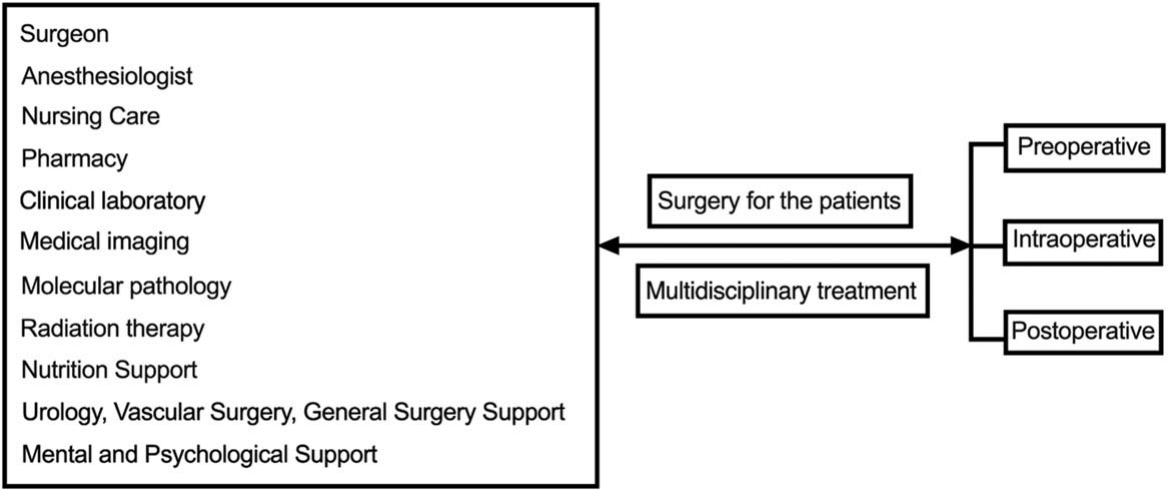
The multidisciplinary treatment of surgery.

As an artificial intelligence (AI) language model, ChatGPT is to provide natural language understanding and generates human-like responses to facilitate interactive and engaging conversations with users. ChatGPT is trained on a vast amount of text data and employs deep learning techniques to learn patterns, understand context, and generate coherent and contextually relevant responses. But, ChatGPT’s responses are based on pre-existing information available in its training data, which has a knowledge cutoff of September 2021. It does not have access to real-time or up-to-date medical knowledge, advancements, or current surgical practices beyond that cutoff date. Therefore, it may not be aware of the latest surgical techniques, guidelines, or emerging research. ChatGPT cannot perform personalized patient evaluations. It does not have the ability to review individual medical histories, physical examinations, or diagnostic test results. Surgical decisions should be based on a comprehensive evaluation of a patient’s specific condition, medical history, imaging studies, and other relevant factors, which require an examination by a qualified healthcare professional. ChatGPT cannot physically examine patients, perform surgical procedures, or provide direct, hands-on guidance during surgeries. Surgical techniques and procedures often require practical skills, precise instrumentation, and real-time decision-making that are beyond the capabilities of an AI language model. ChatGPT’s responses are generated based on the immediate context provided in the conversation. However, it may not have a complete understanding of the specific details or complexities of a surgical case. Providing insufficient or incomplete information may result in incomplete or inaccurate responses. ChatGPT generates responses based on patterns and information it has learned from training data, which may include inaccuracies or biases. While efforts are made to ensure accuracy, there is a risk of misinterpretation or providing incorrect or incomplete information, particularly on specific surgical techniques, procedures, or individual patient situations. ChatGPT does not possess empathy or emotional understanding. It cannot provide emotional support, empathetic responses, or consider the emotional aspects associated with surgery, which can be significant for patients and their families.

Given these limitations, it is important to note that ChatGPT should not be considered a substitute for professional medical advice, diagnosis, or treatment. Surgical decisions and patient care require individualized evaluation, expertise, and a comprehensive understanding of each patient’s unique circumstances. Surgeons and healthcare professionals should rely on their clinical judgment, evidence-based practice, and interdisciplinary collaboration when making surgical decisions and providing patient care.

## Ethical approval

This study does not include any individual-level data and thus does not require any ethical approval.

## Sources of funding

There was no funding for this research.

## Author contribution

X.S.: formal analysis and roles/writing – original draft; J.W.: visualization and roles/writing – original draft; B.W.: data curation and writing – review and editing; X.W.: methodology and writing – review and editing; Z.F.: writing – review and editing; S.W.: visualization and writing – review and editing; K.Z.: project administration and writing – review and editing; D.H.: conceptualization, administration, supervision, and writing – review and editing; Q.Z.: supervision and writing – review and editing.

## Conflicts of interest disclosure

The authors declare that they have no conflicts of interest.

## Research registration unique identifying number (UIN)

Not applicable.

## Data availability statement

The data underlying this article will be shared by the corresponding author on reasonable request.

## References

[1] Vere J Gibson B. Evidence-based medicine as science. J Eval Clin Pract 2019;25:997–1002. 30575209 10.1111/jep.13090

[2] Djulbegovic B Guyatt GH. Progress in evidence-based medicine: a quarter century on. Lancet 2017;390:415–423. 28215660 10.1016/S0140-6736(16)31592-6

[3] Dhawan I Tewari A Sehgal S. Medication errors in anesthesia: unacceptable or unavoidable? Braz J Anesthesiol 2017;67:184–192. 28236867 10.1016/j.bjane.2015.09.006

[4] Hardman B Karamchandani K. Management of anesthetic complications outside the operating room. Curr Opin Anesthesiol 2023;36:435. 10.1097/ACO.0000000000001271 37314173

[5] Marty J Plaud B. Anesthetic process, organization, management and economic issues: the French perspective. Curr Opin Anaesthesiol 2009;22:249–254. 19300244 10.1097/ACO.0b013e32832922a6

[6] Mendes DIA Ferrito CRAC Gonçalves MIR. Nursing Interventions in the Enhanced Recovery After Surgery®: Scoping Review. Rev Bras Enferm 2018;71(Suppl 6):2824–2832. 30540062 10.1590/0034-7167-2018-0436

[7] Nyberg A Olofsson B Otten V. Patient safety during joint replacement surgery: experiences of operating room nurses. BMJ Open Qual 2021;10:e001604. 10.1136/bmjoq-2021-001604 PMC8576473 34750189

[8] Alderson D. The future of surgery. Br J Surg 2019;106:9–10. 30582641 10.1002/bjs.11086

